# Correction to: COVID‑19‑associated Guillain‑Barré syndrome in the early pandemic experience in Lombardia (Italy)

**DOI:** 10.1007/s10072-022-06512-y

**Published:** 2022-11-25

**Authors:** Filippo Martinelli‑Boneschi, Antonio Colombo, Nereo Bresolin, Maria Sessa, Pietro Bassi, Giampiero Grampa, Eugenio Magni, Maurizio Versino, Carlo Ferrarese, Davide Zarcone, Alberto Albanese, Giuseppe Micieli, Carla Zanferrari, Antonio Cagnana, Claudio Ferrante, Angelo Zilioli, Davide Locatelli, Maria Vittoria Calloni, Maria Luisa Delodovici, Mattia Pozzato, Valerio Patisso, Francesco Bortolan, Camillo Foresti, Barbara Frigeni, Stefania Canella, Rubjona Xhani, Massimo Crabbio, Alessandro Clemenzi, Marco Mauri, Simone Beretta, Isidoro La Spina, Simona Bernasconi, Tiziana De Santis, Anna Cavallini, Michela Ranieri, Elisabetta D’Adda, Maria Elisa Fruguglietti, Lorenzo Peverelli, Edoardo Agosti, Olivia Leoni, Andrea Rigamonti, Andrea Salmaggi

**Affiliations:** 1 Neurology Unit, IRCCS Fondazione Ca’ Granda Ospedale Maggiore Policlinico, Via Francesco Sforza 35, 20122 Milan, Italy; 2 Department of Health Sciences, University of Milan, Via Antonio di Rudinì 8, 20142 Milan, Italy; 3 Membro Direttivo Nazionale SNO, Polo Neurologico Brianteo, Seregno, MB Italy; 4 U.O. Neurologia Ospedale Giovanni XXIII, Bergamo, Italy; 5 U.O. Neurologia, Ospedale San Giuseppe, Milan, Italy; 6 U.O. Neurologia, Ospedale Sant’Anna, Como, Italy; 7 U.O. Neurologia Poliambulanza, Brescia, Italy; 8 Università Dell’ Insubria U.O. Neurologia Ospedale Di Varese, Varese, Italy; 9 Università Degli Studi Milano Bicocca, U.O. Neurologia, Ospedale San Gerardo, Monza, Italy; 10 U.O. Neurologia, Ospedale Sant’Antonio Abate, Gallarate, VA Italy; 11 U.O. Neurologia, IRCCS Istituto Clinico Humanitas, Milan, Italy; 12 U.O. Neurologia, Fondazione Mondino, Pavia, Italy; 13 U.O. Neurologia, Ospedale Vizzolo Predabissi, Vizzolo Predabissi, MI Italy; 14 U.O. Neurologia, Ospedale Maggiore, Crema, CR Italy; 15 U.O. Ospedale Policlinico Ponte San Pietro, Ponte San Pietro, BG Italy; 16 U.O. Neurologia Ospedale Maggiore, Lodi, Italy; 17 Università Insubria, U.O. Neurochirurgia Ospedale Di Varese, Varese, Italy; 18 Membro Direttivo Nazionale SNO, Rome, Italy; 19 Membro Direttivo Regionale Lombardo SNO, Milan, Italy; 20 U.O. Osservatorio Epidemiologico Regionale, Struttura Epidemiologia E Valutazione Delle Performance, Milan, Regione Lombardia Italy; 21 U.O. Neurologia, Ospedale Manzoni, Lecco, Italy; 22 Coordinatore SNO Lombardia, U.O. Neurologia, Ospedale Manzoni, Lecco, Italy

**Correction to** **:** **Neurological Sciences (2022)**

**https://doi.org/10.1007/s10072-022-06429-6**

The above article was published with error. The affiliation address of the authors Alberto Albanese and Tiziana De Santis has been modified to: “U.O. Neurologia, IRCCS Istituto Clinico Humanitas, Milan, Italy”.

The Original article has been corrected.

